# Similar skills, different frames: a thematic analysis exploring conceptualizations held by community-based organization practitioners and academics regarding skills to use evidence-based interventions to address cancer inequities

**DOI:** 10.1186/s43058-023-00472-w

**Published:** 2023-07-26

**Authors:** Shoba Ramanadhan, Jennifer L. Cruz, Maggie Weese, Natasha Naveed, Shinelle Kirk, Madison K. Rivard, Judi Kirk, Albert Whitaker, Karen Peterson, Arthur Eisenkraft

**Affiliations:** 1 Department of Social and Behavioral Sciences, Harvard TH Chan School of Public Health, 677 Huntington Ave, Boston, MA 02115 USA; 2 University of Massachusetts Boston, 100 William T. Morrissey Blvd, Boston, MA 02125 USA; 3 Conservation Law Foundation, 62 Summer St, Boston, MA 02110 USA; 4 Boys and Girls Club of Worcester, 65 Boys & Girls Club Way, Worcester, MA 01610 USA; 5 American Heart Association, 300 5Th Ave, Waltham, MA 02451 USA; 6 St. Mark Congregational Church, 200 Townsend St, Boston, MA 02121 USA; 7 Tufts Medicine, 800 District Avenue, Suite 520, Burlington, MA 01803 USA

**Keywords:** Evidence-based interventions, Community-based organizations, Capacity-building, Training, Professional development, Practitioners

## Abstract

**Background:**

Community-based organizations (CBOs) are critical partners in delivering evidence-based interventions (EBIs) to address cancer inequities. However, CBO practitioners do not typically have access to opportunities to build the necessary capacity (skills, knowledge, motivation, and resources) for using EBIs. Although capacity-building interventions can offer a solution, inconsistent definitions and measurements of capacity limit the ability to develop and evaluate such efforts. We explored how and why conceptualizations of core skills for EBI use differ between practitioners and academics addressing cancer and other health inequities. We anchored the inquiry with a commonly used set of target skills for EBI capacity-building efforts.

**Methods:**

The study was conducted by an interdisciplinary team of academic researchers and CBO practitioners. We gathered data through semi-structured, hour-long interviews with practitioners and academics working to address cancer and other health inequities (*n* = 19). After hearing a brief vignette about a CBO addressing cervical cancer inequities, participants considered a widely accepted list of skills for EBI use that included assessing needs, engaging stakeholders, and selecting, adapting, implementing, evaluating, and sustaining the EBI. We used a team-based, reflexive thematic analysis approach grounded in critical and constructivist perspectives.

**Results:**

Overall, the original list resonated with practitioners and academics and they added new skills to the list (cultural humility and systems change). Practitioners’ responses described skills from the reference point of addressing broader community needs and context and achieving change over the long term, emphasizing aspects of health promotion in their descriptions. Academics offered a mix of perspectives, with some focused on addressing community needs (and related flexibility regarding EBIs) but more emphasized skills needed to deliver a specific EBI to achieve a focused set of health and equity outcomes.

**Conclusions:**

There is a significant opportunity to leverage complementary expertise and perspectives held by practitioners and academics addressing cancer inequities. However, the different frames utilized suggest proactive efforts will be required to find alignment across groups, particularly in valuing diverse contributions and identifying relevant outcomes of interest for each group. Such alignment is critical to designing effective capacity-building interventions and supporting the routine utilization of EBIs to address cancer inequities.

**Supplementary Information:**

The online version contains supplementary material available at 10.1186/s43058-023-00472-w.

Contributions to the literature

- Insufficient capacity to use evidence-based interventions to address cancer inequities constrains the health impact of community-based organizations (CBOs).
- To address gaps in the science and practice of capacity-building for CBOs, a team of academics and CBO practitioners explored the perspectives of CBO practitioners and academics regarding core skills needed to use evidence-based interventions.
- Our study identified overlapping perspectives on what the lists should include and critical differences regarding the lenses through which capacity-building targets are viewed, which must be bridged to increase the relevance and impact of capacity-building offerings.

## Background

Community-based organizations (CBOs) are well-positioned to integrate the best available research evidence, local expertise, and community priorities to improve health [1–4]. Routinizing the use of evidence-based interventions (EBIs) in these settings offers a vital opportunity to advance health equity as CBOs have rich reach and trust among populations that are (a) subject to structural forces that create and exacerbate inequities and (b) ineffectively served by conventional public health and healthcare institutions [4–6]. However, EBI use in CBOs is limited due to insufficient training, skills, and support to use EBIs, constrained time and resources, and difficulty sustaining programs [7–11]. Challenges in using EBIs are exacerbated for CBOs working with underserved communities as they face heightened constraints on funding, staffing, and other resources [6, 9]. This highlights the need to design and develop capacity-building strategies to support EBI use among CBO practitioners working with underserved communities.

Capacity-building for EBI use has increased the adoption and implementation of EBIs among local health department staff, policymakers, and public health practitioners in community settings [7, 12, 13]. These programs typically target knowledge, skills, motivation, and resources needed to use EBIs and often offer training, technical assistance, manuals/tools, and other support [14]. However, three limitations of the current knowledge base must be addressed to support the development and evaluation of effective capacity-building interventions in CBO settings. First, the literature emphasizes capacity-building for EBI use in clinical, health department, and mental health agency settings [7], which have different organizational structures, resources, and staff types than CBOs, which are typically board-led nonprofits delivering services in coordination with community stakeholders [15]. CBOs are often engaged in partnership-based delivery of services, e.g., the engagement by YMCAs in referral programs with clinical sites or connecting with housing authorities to support clients [16]. Second, researchers operationalize capacity-building in diverse and sometimes incompatible ways [7]. A recent scoping review of 99 CBO capacity-building studies found a tremendous diversity of definitions (and capacity-building targets) and limited use of validated measures [17]. Third, practitioners identify numerous ways in which capacity-building interventions are mismatched with implementation settings, practitioner expertise, and community assets and needs. There is a clear need to center history, structural determinants of health, and community needs and resources to advance capacity-building efforts [18, 19], but it is unclear how best to do this. Finally, models of EBI use in community settings have primarily been driven by academic researchers and national health organizations such as the CDC [2, 20].

To address these gaps in the literature, we conducted an inquiry to understand how practitioners and academics may see core EBI skills anchored in a popular list of capacity-building targets. The standard list builds on the Cancer Prevention and Control Research Network's curriculum, a national standard promoted by the CDC and other national health organizations. This list included engaging stakeholders; assessing needs; and selecting, adapting, implementing, evaluating, and sustaining EBIs [20, 21]. We started with skills with the expectation that findings would support the explication of other domains of capacity (knowledge, motivation, and resources). Additionally, we grounded the exploration in an example of an opportunity for implementation science to advance health equity: cervical cancer. There are existing technologies to detect cancers early and disproportionate disease burden experienced by systematically excluded populations, including some racial and ethnic minority groups, those of lower socioeconomic status, and rural residents [22, 23]. At the same time, CBOs can play an essential role in increasing demand and reducing barriers to services, which the US Preventive Services Task Force recommends to address service gaps [4, 6, 24]. We sought to understand how CBO practitioners and academics conceptualize the core skills needed to use EBIs to address health inequities and how/why these conceptualizations may differ.

## Methods

### Project background and study design

Data for this study came from the Community Links Project. They were collected as part of formative work to support the development of measures related to the capacity for CBO practitioners to use EBIs with marginalized populations. We gathered data using semi-structured interviews with two groups of participants working to address health inequities: practitioners and academic researchers. We asked common questions for both groups and allowed flexibility to follow individual participants' experiences and unanticipated areas of inquiry [25]. We utilized constructivist and critical perspectives, recognizing that study insights would be co-created by practice-based experts and the study team, would reflect our values and positions, and must be applied to address inequities [26]. The study focused on practitioners' and academics' reactions to a standard list of EBI skills from the Cancer Prevention and Control Network's Putting Public Health Evidence in Action curriculum, a national standard [20]. We added sustainability to reflect the current move in the field of implementation science to focus on this concept [21]. The final list included engaging stakeholders (connecting with communities affected by the health issue of interest and individuals/organizations that will deliver evidence-based services; assessing needs (characterizing the health issue and goals of interest); selecting the EBI (finding an intervention that fits needs and resources); adapting the EBI (making changes to increase impact and relevance); evaluating the effort (assessing the impact of the implementation effort); and sustaining the EBI (integrating the intervention into the organization if appropriate).

### Team composition

The study team has expertise in behavioral science, implementation science, cancer inequities, participatory research, and education/professional development and includes members of racial, ethnic, and geographic groups experiencing cancer inequities. The lead investigator [SR] has 15 years of experience supporting EBI utilization in CBOs serving marginalized populations. Other team members included a doctoral student with experience in implementation science and cancer equity [JC], a master's student [NN] and professor [AE] with expertise in education and professional development, and two master's students with expertise in community health [CC and MW]. The team also includes three community leaders with rich practice-based expertise with EBIs in CBOs to advance health equity. We engaged these leaders as advisors, using a consultative model to balance gathering their insight and maintaining a reasonable burden [27]. All three are co-authors of this paper [JK, AW, and KP].

### Participants

The study balanced input from practitioners and academic researchers (Table 1). The first group included practitioners from and leaders of CBOs (referred to throughout as practitioners). Inclusion criteria: adults; working in a CBO in the USA; 5 + years of public health practice experience; have used one or more EBIs; and working to address cancer and/or other health inequities. We recruited them through the study team's professional networks and Internet searches for cancer control partnerships and local nonprofits addressing cancer inequities. The second group included academic researchers. Inclusion criteria: adults; working in the USA; 5 + years of research experience; addressed cancer inequities in their work; and conducted research on EBIs or implementation science. We recruited participants through the study team's professional networks and by searching the NIH REPORTER and PubMed websites. For both groups, we attempted to maximize the diversity of participants by region and communities served to attain sufficiently information-rich cases [28]. We used interview summary templates to capture details after each session and reviewed these regularly to reduce the risk of oversight or missing voices. The number of participants for this exploratory study was a function of information power and reflected the focused aim, specificity of the sample, emphasis on existing theory, rich dialogue, and goals of case-specific and cross-case analysis [29].

**Table 1:** Participant characteristics (*n* = 19)

	**Academics** (*n* = 11)	**CBO practitioners** (*n* = 8)	**Total** (*n* = 19)
**Region**			
Northeast	1	5	6
South	6	1	7
Midwest	2	2	4
West	2	0	2
**Populations served (multiple selections permitted)**			
Black or African American	5	4	9
Latinx/Hispanic	5	3	8
Asian	0	2	2
American Indian or Alaska Native	0	1	1
Pacific Islander	0	0	0
Rural	4	1	5
LGBTQ +	2	6	8
Low-income	5	6	11
**Years of experience** (mean, standard deviation)	25.6 (8.1)	16.5 (5.9)	21.7 (8.2)

### Data collection

Interviews were conducted from February to April 2021. Lead interviewers were experienced qualitative researchers with doctoral degrees in public health and social and cultural psychology. Interviews were conducted using a videoconference platform with a telephone call option. The interviews were scheduled for 1 h and averaged 43 min (range: 34–59 min). Four participants (two practitioners and two academics) were known to members of the study team in advance, and the rest were new contacts. Study procedures were explained, and informed consent was obtained before the interview commenced. Participants received a $50 gift card as a token of appreciation.

Interviews followed a semi-structured interview guide, including a 1-min vignette describing a CBO applying for a grant to use an EBI to increase cervical cancer screening among Latinas. We asked participants to consider the skills practitioners would need to use the EBI successfully and displayed on a shared screen a list of common evidence-based public health skills: engaging stakeholders; assessing needs; and selecting, adapting, implementing, evaluating, and sustaining EBIs [20, 21]. Participants were prompted to offer additions, edits, and removals. Interviews were audio-recorded and professionally transcribed. The Institutional Review Board at Harvard University designated this study exempt from review.

### Data analysis

We employed a team-based, reflexive thematic analysis approach for this study, guided by the work of Braun and Clarke [30, 31]. After each interview, the study team compiled notes using a prefigured interview summary table that included key domains from the interview guide, emerging insights, key takeaways, and notes about context. The initial codebook included deductive codes from the interview guide and inductive codes from the interview summaries and transcript reviews. The analytic team met to review, modify, and finalize the codebook. Two study team members (JC and NN) independently coded all transcripts and met weekly to compare coding and address disagreements. Coding for this manuscript prioritized data related to reactions to the standard skills list and relevant information about those expected to employ EBI skills and the contexts within which they work. After coding was complete, team members (JC, NN, and CC) summarized selected codes, and the full analysis team met regularly to review summaries, identify themes, and select representative quotes. Practitioners on the team reviewed early results, prompting revisions of interpretations and later advanced arguments through manuscript development. We used the standards for reporting qualitative research checklist [32] to ensure all relevant details were communicated in the manuscript (Supplemental File 1).

## Results

Our data highlight a few patterns regarding different conceptualizations or areas of emphasis between practitioners and academics. First, we found much common ground regarding reactions to the core EBI skills. Second, we identified essential differences in the lenses through which academics and practitioners viewed EBI skills, with practitioners focusing more on broad community needs/transformational goals and academics focusing more on the EBI. Below, we present how skills from the original list were described, additions to the list, and the underlying differences in perspectives brought to bear by practitioners and academics.

### Views of core skills

The original skills list resonated with both groups, with several additions or extensions per participant and no suggestions to drop any original skills. Participants described opportunities to orient each of the skills towards equity, e.g., using process evaluation to monitor reach and ensure that it was equitable across groups. They also described a common expectation that skill acquisition could increase equitable access to and use of resources among implementation partners. Many participants highlighted the need to address cancer inequities with EBIs while ensuring other inequities were not created or exacerbated. Others noted that while skills can be built, the impact will be limited if broader issues around insufficient funding for services or accessibility of EBIs are not addressed. One practitioner felt that capacity for EBI use could not be built among CBO staff, but this was the only example of this divergent view. Many of the skills were also linked to participatory processes connecting EBI delivery with community and partner engagement and developing multi-level solutions to improve health equity. On a related note, the skills were often described as inherently linked and used in combination. The following offers an example of both participatory approaches and the integration of skills:

The partnerships in communities or with communities are fundamental to being able to identify and appropriately assess needs. It’s really hard to assess needs and then to try to find partners who are willing to also prioritize those needs given extant data… There’s often a mismatch between what data says and what communities prioritize. – Interview 16 (academic)

#### Engaging partners

Discussions of partner engagement centered on two main groups: community members and organizational partners. Regarding community engagement, one set of responses emphasized engaging community members in unique ways. Practitioners and academics described engaging service recipients and those in the broader social system to ensure community knowledge influenced EBI implementation efforts. These community members could be engaged either directly or through trusted individual or organizational intermediaries; practitioners framed engagement as beneficial for the planning and implementation processes, as described here:

Even in identifying these evidence-based interventions, I think most places should take a step back to ask that community what would work for them… the length, the intensity, the duration… to say, hey, look, is this going to work, and… the odds of them being successful in the intervention, it's not stacked against them. – Interview 18 (practitioner)

Practitioners and academics also described the importance of gaining buy-in and support for the EBI effort. In this way, skills for relationship development could offer the necessary legitimacy and connections in the community to support EBI implementation. Finally, some practitioners described community engagement as an opportunity to contribute to the community, whether through workforce development or redirection of resources:

So being able to provide [youth] with payment to participate in a program and then learning the skills to then teach their peers– it’s a matter of like workforce development skills that they’re gaining in general, and then they’re also gaining an opportunity to like potentially save money or support their family which we’ve found is really helpful…With our parent workshops and our youth facilitating a lot of our work, it allows them to gain leadership skills. So we also focus on parent advocacy and how we can get parents and youth ready to advocate in their community. – Interview 17 (practitioner)

Regarding organizational partners, practitioners, and academics emphasized skills for strategic partner selection, particularly in terms of aligned goals. This was offered as a direct contrast to seeking to partner with the organization that usually gets funds or is most visible but may not be as well-connected to groups experiencing inequities. Practitioners and academics described the utility of seeking non-traditional partners offering a unique synergy, e.g., working with a housing authority. The following example of offering outreach content as part of English as a Second Language class staffed by volunteers illustrates this nicely:

We’re working with different [English as a Second Language] ESL providers to develop a health curriculum… So, then you create a supporting environment and people feel safe, people don’t feel stigmatized when they were introduced to different disease topic... Another innovative idea is that we wanted to frame this as a community service learning opportunity for medical students, for companies that wanted to do community service… it’s win, win, win for everybody. – Interview 1 (practitioner)

Another set of suggestions related to skills in developing, managing, and sustaining partnerships. Practitioners and academics emphasized the skills needed to partner with organizations and individuals with differing norms, cultures, and ways of working from their own. Examples ranged from conceptual (e.g., understanding partner motivations) to practical (e.g., identifying communication preferences of different partners). These skills were often linked to participatory decision-making and engagement processes. Participants described a need to engage with a diversity of actors to generate agreement and coordinated action through collaborative decision-making:

If we want to do something community-wide, then those collaboration skills and partnership forming skills and participatory decision making with diverse audiences and diverse stakeholders becomes so much more important. Because then you’re trying to get multiple organizations working together and doing things in the community. So I think that becomes increasingly important as you move up levels of the socioecological model, just because you have – you just have more diversity and more partners. – Interview 10 (academic)

Often bridging agents have one foot in the organization and one foot in the community. So and I can think prototypically, like somebody who grew up in a marginalized community and has been able to take advantage of and get an education that affords them the opportunity to be in a position of working in a CBO. Right? So they know … the moms and the grandmas and the aunties in the community and can speak their language. And then they come in into the CBO and talk about outcomes and evaluation and evidence-based practices, and so they code shift or whatever… being able to move between the community and the agency is its own, I think, profound set of skills. – Interview 5 (academic)

#### Assessing needs

Practitioners and academics discussed skills for incorporating structural factors that drive inequities into needs assessments, e.g., transportation issues or discrimination. Many participants described the need to examine the context in which individuals receive services as critical for using EBIs to advance health equity. The emphasis on system-level issues is exemplified by this quote:

I would say making sure that as you are assessing the need that your data approach is a holistic approach that gets you to also get to some of the why, which is, I think, where your barriers lie, but then also taking a step further into saying, well, if that is a barrier, why? What makes it be such a significant challenge for this community versus another community? – Interview 18 (practitioner)

One academic described the application of equity-focused frameworks as valuable support for inclusive needs assessments. These assessments were often linked to the acknowledgment that a given EBI cannot address all of the upstream concerns affecting a given community:

People really need to understand racism and classism, sexism, heterosexism, and able-ism. If you’re going to address health equity and you don’t have a good understanding of the privileges that exist based on social determinants, you’re not going to be as effective. So I do think that kind of training is helpful if you want to address health equity. – Interview 10 (academic)

Additional skills related to needs assessments were described concerning community relationships. Practitioners noted a need to ensure that the burden or depth of the assessment would not damage relationships and instead could be used to support community engagement. One academic noted that needs assessments could provide an opportunity to engage community members early in the EBI process. Last, the requirement to assess needs using participatory processes was highlighted by many participants:

And the more you’ve involved your focal community and the more you’re focused on building capacity in that focal community, the more you’re gonna be responsive to their self-needs. And so it might not just be about cervical cancer screening, but also doing more than just cancer focused work because I think a lot of the time the more pressing needs are the ones the focal community’s gonna bring up – Interview 10 (academic)

#### Selecting, adapting, implementing, and sustaining EBIs

While these are separate skills, much of the content was similar a) across groups and b) to the base model of skills presented. Linked to selection and adaptation, many participants emphasized the need for skills in identifying, adapting, and implementing EBIs that meet the needs of marginalized communities and community members. They emphasized the importance of building bridges between the EBI at hand and the needs and priorities of the community, e.g., by integrating local concerns into the EBI or finding other opportunities for alignment. Participants also linked skills related to selecting, adapting, implementing, and sustaining EBIs as fundamentally linked to community engagement:

During the implementation process, make sure that there’s frequent and just ongoing communication. And whether that’s working, is it not working. But I think even beyond that to say, hey, look, when we came to you six months ago, this is kind of where we were. Are there some other issues that … have come to light, and is that also something we can help you with? – Interview 18 (practitioner)

For adaptation, much of the content centered on common areas of focus relating to increasing the impact and relevance of the EBI. Additionally, practitioners and academics discussed the need for communication skills to support EBI use. Several practitioners emphasized the ability to adapt communication (e.g., style, frame, language complexity) for diverse audiences served, especially for groups whose needs are unmet by broadly accessible materials. Another aspect of communication highlighted by practitioners was related to using diverse channels for diverse audiences. Practitioners noted a need to use various media types and communication vehicles, from Instagram and Facebook to town halls and community meetings. This skill was tied both to outreach related to the EBI and communication with partners and stakeholders:

Whether it’s an infographic, whether it’s a brief update, a presentation, an article in the newspaper that they read, or whatever their news source is, as to what you’re doing, and how it’s going… Keeping them engaged and reporting back to stakeholders is key to success. – Interview 8 (practitioner)

Academics primarily discussed developing communication skills to support linking the project with partners and community members. Other areas of focus included the customization of EBI materials for community member needs and the use of a wide range of communication strategies, ranging from social media to attending town halls and sharing information via local radio programs.

#### Evaluation

Almost all academics discussed skills related to evaluation. They highlighted the need for skills related to monitoring, particularly in the context of examining impacts on health equity and taking corrective action as needed. Participants also discussed costs as an important component of evaluation, including assessing the costs of collecting monitoring and evaluation data that CBOs will incur. Similar discussions focused on the results of evaluation activities. Participants noted that skills were needed to ensure findings were presented back in a manner accessible to stakeholders. This highlighted the need to demonstrate how collected data would be used for research and local action.

#### Cultural awareness/humility (addition)

Practitioners and academics highlighted cultural awareness or humility skills as critical for EBI implementation. Almost all practitioners discussed this as an additional skill, but only a few academics discussed this skill, and one academic suggested that this could not be taught. Practitioners emphasized the importance of skills related to identifying and decreasing implicit bias, helping clients and community members address stigma, and understanding the roots of health inequities. They offered examples of the need for cultural awareness concerning the country of origin, race, ethnicity, immigration status, sexual orientation or gender identity, and religion. Many responses emphasized a view of clients and community members grounded in respect and empowerment:

Having somebody that can really listen. Really listen and not try and solve the problem, but try and say, okay, this is what I’m hearing, does anybody have any suggestions for this person? And it doesn't mean that you can’t be included in that. But these evidence-based programs are really designed to empower people, not to just sit there and tell them what to do. – Interview 2 (practitioner)

Another response related to cultural awareness or humility connected with group membership and lived experience of practitioners. Participants highlighted the importance of practitioners leveraging the knowledge of communities based on shared identities as part of engaging with diverse audiences:

I would say one of the unique pieces with working in a community-based organization is that oftentimes we…live in the community or grew up in the community, and having that community connection can have great impact. – Interview 17 (practitioner)

Among academics, a common sentiment was the need to understand enough about the clients’ or community members’ lives to place EBI content in context. The need for empathy and an understanding of the community was described as critical:

I really can’t put enough emphasis in skills on the cross-cultural piece because it’s not gonna work if you have some person who is talking to a 60-year-old [group] woman who just doesn’t have a clue about their life. So some maturity and life experience. – Interview 13 (academic)

#### Systems change (addition)

Although not common, a few participants discussed skills related to systems change and the ability to address “upstream factors” as necessary as part of addressing health inequities. Others mentioned that all of the skills of the initial list should be considered in the context of systems change. One academic offered a perspective by which skills for system change would support the use of the implementation effort as an opportunity to reshape public health systems:

Whenever you bring a program into a setting, it sometimes challenges the underlying system assumptions about what are the roles people should play? What are the values?... And sometimes the power of these evidence-based interventions is not in sustaining the program itself, but in actually shifting the system, the very system that’s supporting that program… [The systems orientation] is really highlighting and that maybe I would say overvaluing the perspective of the population that you’re trying to serve. – Interview 19 (academic)

Others noted that systems change work might involve examining power hierarchies that impact implementation efforts:

Recognizing where inherent power differentials are between people. And also in meetings themselves, I mean, paying attention to … how people perceive themselves on any type of hierarchy. So I’ll notice, oh, the people with the doctor degrees say a lot, and the people that have nursing degrees, or no degree at all, or high school, say less. And so then the question becomes, how do you try to rebalance some of that power in your implementation initiative. – Interview 11 (academic)

### Differences in focal points for practitioners vs. academics

While there were many commonalities in how practitioners and academics mapped needed EBI skills onto a standard list, their discussions of skills had different focal points. Practitioners tended to focus on skills as part of serving the individuals and communities receiving programming or services, whereas academics often discussed skills in the context of EBI delivery. An example of discussing needs assessment illustrates this perspective:

[You need to make] sure that as you are assessing the need that your data approach is a holistic approach that gets you to also get to some of the why, which is, I think, where your barriers lie, but then also taking a step further into saying, well, if that is a barrier, why? What makes it be such a significant challenge for this community versus another community? – Interview 18 (practitioner)

Practitioners often included general skills related to broad goals of health promotion, e.g., moving community members towards their long-term goals as part of EBI use. An illustration of that perspective was skills to assess and address community members’ comfort with and trust in services delivered by healthcare and public health organizations, both related to the EBI and other health needs.

There was a mix of viewpoints among academics, as some described skills in the context of flexibility in terms of issue and EBI selection that could integrate with community goals. Others were focused more on a selected EBI and thus considered the alignment with longer-term goals as a determination of whether or not implementation of that EBI was appropriate. For needs assessment, most academics emphasized skills to ensure the EBI reached and benefited populations of interest:

It also gets you on your way of understanding the context as those people see it… [And] it’s unique to how they're going to implement… the evidence based intervention.- Interview 11 (academic)

## Discussion

The study explored whether and how practitioners and academics differ in conceptualizing core skills for EBI use to address cancer inequities, anchoring our exploration with a widely-accepted list of core skills. We found that the original list of skills resonated broadly across practitioners and academics, with some new skills added (e.g., cultural awareness) and existing skills extended (e.g., considering them with a participatory lens). At the same time, different focal points between practitioners and academics regarding how they considered EBI skills suggest a critical need to generate alignment and support the co-creation of capacity-building interventions. In this way, the complementary expertise brought to bear by practitioners and academics can advance the use of EBIs to address health inequities.

Perspective is critical when considering the distinctions between practitioners’ and academics’ views of core skills. Bronfenbrenner’s ecologic systems theory offers a useful parallel, given its attention to multi-level influences on behavior related explicitly to an identified group of focus [33]. In that sense, all participants described the goal of improving health among service recipients but with different implicit referent points. Practitioners described skills in relation to community members, e.g., the extent to which EBI implementation could support community members’ goals beyond the focal health issue. Some academics shared this view, but the bulk of conversations related to skills specific to the delivery of an EBI and broad goals linked to EBI delivery, e.g., changing systems. This is consistent with the literature suggesting that academics typically rely on a technology transfer model, which focuses on the EBI and the system’s needs concerning that solution [34]. In contrast, CBOs tend to take a holistic view in their work [35]. As highlighted by Trickett, capacity-building often attends to building for an EBI. Still, if the long-term goals include using research evidence in community settings and broader community advancement, the work must attend to other practice and community goals [36]. The different focal points also reflect the institutions and incentives for each group of professionals, with CBO practitioners and CBOs oriented towards service delivery and community goals [15]and academics who were implementation scientists oriented towards the advancement of knowledge and practice related to the integration of EBIs into routine care [37, 38]. This may seem obvious, but the differing perspectives require the thoughtful design of capacity-building interventions to acknowledge and bridge these differences. Another important potential capacity-building challenge relates to the emphasis placed by practitioners on concepts of health promotion, which supports community members to influence the factors that impact their health and allows them to achieve their goals [39, 40]. Most of the public health workforce does not have formal training in public health [2]. Thus, if these concepts are implicit in other domains in models of evidence-based public health [20, 41] but are not core training targets, practitioners may miss out on the opportunity to build needed skills. For practitioners who do have these skills, this should provide additional opportunities related to professional advancement and compensation.

**Fig. 1:**
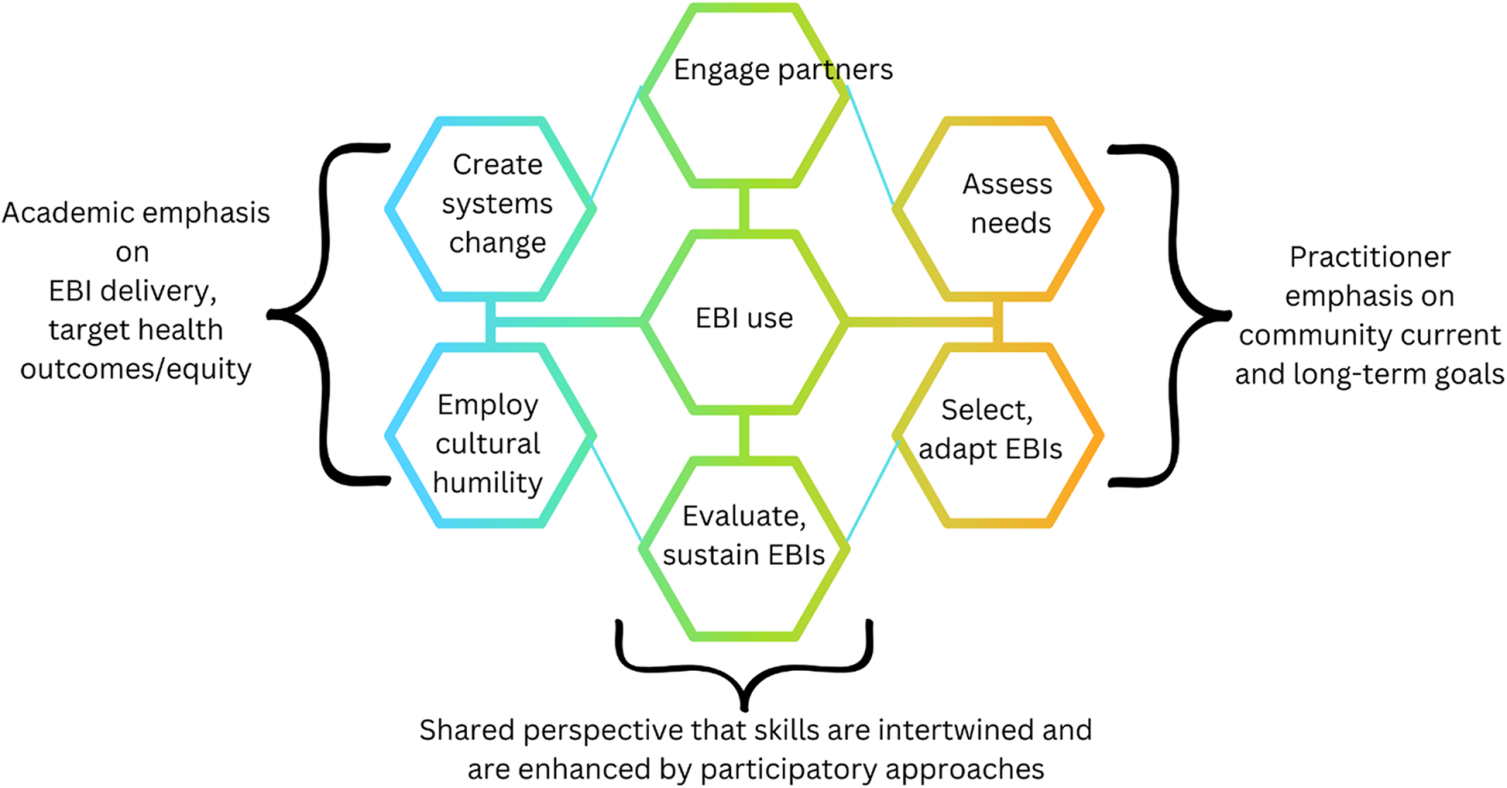
Conceptualizations of core skills for EBI use in CBOs by practitioners and academics addressing cancer inequities

The opportunity for alignment may come about through participatory processes highlighted by many participants. Such approaches offer the opportunity to align capacity-building for EBI use with the priorities, resources, and current activity CBOs are already engaged in. Participatory approaches grounded in emancipatory traditions also include efforts to create transformational change in social systems [42], which connects with the focus on systems change, as well as practitioner goals related to addressing the long-term goals of community members. Figure 1 offers a visual summary of the similarities, differences, and aligned understanding among practitioners and academics.

Another opportunity for alignment comes from considering positionality, examining how different experiences and perspectives may influence assumptions and understandings of EBI skills, perspectives that may be missing, how practitioners and academics are “read” by those they interact with, and dynamics of privilege or marginalization that influence EBI implementation [43]. This links with the emphasis many participants placed on ensuring that EBIs are implemented by individuals and organizations deeply connected to the populations of interest, particularly given the focus on health inequities. This type of intentional staffing relates to the importance of leveraging practitioners' local, client-based, and practice-derived expertise, consistent with evidence-based public health concepts that connect research evidence with practice-based expertise, community needs and preferences, and information about the local environment [41, 44]. However, these points are in tension with the current literature highlighting practitioners’ concerns about what evidence “counts” in public health and practice systems, the discounting of practice-based expertise, and the patterns in which academics drive the knowledge production agenda [45–47].

We place our findings in the context of limitations and strengths. One limitation is that we focused on individuals working with cancer and other health inequities and offered a vignette focused on cervical cancer; thus, there may be some patterns specific to this topic. However, given the emphasis on general capacity versus EBI-specific capacity and the rigor of the analytic process, we expect the findings to be transferable to other settings. Another potential limitation of the study is that we prompted practitioners and academics to review and react to one list of skills for EBI utilization, which may have constrained their answers. This was an intentional decision as we wanted to draw comparisons to currently accepted lists of skills. Finally, while our ultimate goal with this body of work is to redesign capacity-building interventions, all of our participants had experience with EBIs as a function of the inclusion criteria. That may have an impact on capacity-building efforts for practitioners new to EBIs. Several strengths outweigh these limitations—first, we privileged expertise from practitioners and academics equally. Incorporating diverse expertise is expected to offer a more comprehensive and practical result than we could have achieved with one group alone. Second, our focus on individuals addressing inequities addresses the current gap in the implementation science literature, which does not sufficiently attend to research focused solely on implementation among populations experiencing inequities [48], which reduces the utility of the solutions developed. Third, the inclusion of CBO practitioners and academics on the research team allowed us to draw from diverse relevant experiences and perspectives.

## Conclusions

The findings from this study suggest a rich overlap in the conceptualization of core skills for EBI use and important differences in the frames and reference points used by practitioners and academics. By understanding and valuing differential frames from the outset, practitioners and academics can integrate their complementary expertise and perspectives to advance capacity-building for EBI utilization in marginalized communities. Open discussions to find alignment of EBI-related and broader goals, as well as identification of and respect for the range of skills needed to implement EBIs successfully, will offer opportunities to ensure that capacity-building interventions are relevant and high-impact for CBO practitioners, a critical set of players to advance health equity.

## Supplementary Information

**Additional file 1.**

## Data Availability

The datasets generated and/or analyzed during the current study are not publicly available as participants' privacy and confidentiality would be compromised, but are available from the corresponding author on reasonable request.

## Abbreviations

CBO: Community-based organization
EBI: Evidence-based intervention
